# Effects of age and experience on contest behavior in the burying beetle, *Nicrophorus vespilloides*

**DOI:** 10.1093/beheco/art101

**Published:** 2013-11-08

**Authors:** Victoria E. Lee, Megan L. Head, Mauricio J. Carter, Nick J. Royle

**Affiliations:** ^a^Centre for Ecology and Conservation, College of Life and Environmental Sciences, University of Exeter, Cornwall Campus, Treliever Road, Penryn, Cornwall TR10 9EZ, UK and; ^b^Division of Evolution, Ecology and Genetics, Research School of Biology, The Australian National University, Acton, ACT 0200, Australia

**Keywords:** age, contest behavior, fighting, male competition, Nicrophorus vespilloides, social experience, terminal investment, winner–loser effect.

## Abstract

Contest behavior forms an important part of reproductive investment. Life-history theory predicts that as individuals age and their residual reproductive value decreases, they should increase investment in contest behavior. However, other factors such as social experience may also be important in determining age-related variation in contest behavior. To understand how selection acts on contest behavior over an individual’s lifetime, it is therefore important to tease apart the effects of age per se from other factors that may vary with age. Here, we independently manipulate male age and social experience to examine their effects on male contest behavior in the burying beetle *Nicrophorus vespilloides*. We found that social experience, but not age, influenced male contest behavior but that these changes in behavior did not alter contest outcomes. Male size (relative to his opponent) was overwhelmingly the most important factor determining contest outcome. Our results suggest that in systems with high variation in fighting ability among males, there may be little opportunity for selection to act on factors that influence contest outcomes by altering motivation to win.

## Lay summary

Aggression and likelihood of winning contests are expected to change as a male ages. We test this idea in burying beetles, a species which competes over small mammal carcasses as a breeding resource. We find that male size relative to his opponent is far more important in determining contest outcome than any effects of age or social experience.

## INTRODUCTION

Animals will fight over resources if access to those resources is a constraint on fitness (Briffa and Hardy 2013). Such contests over critical resources are remarkably widespread among animal taxa, and the outcomes of these contests often have profound consequences for fitness (Wong and Candolin 2005; Eggert et al. 2008).Therefore, individuals are under intense selective pressure to behave in a way that maximizes long-term success when making decisions about who, when, and how to fight (Rutte et al. 2006).

Much research has focused on the factors underlying variation in aggression and likelihood of winning contests. Aggressive behavior and contest outcome are dependent on resource holding potential (RHP, Parker 1974), which has been shown to be influenced by 2 primary factors. First, RHP is strongly correlated with asymmetries in the fighting ability of opponents, which are often determined by body size (e.g., burying beetles, *Nicrophorus spp.*—Otronen 1988; stalk-eyed flies, *Teleopsis dalmanni*—Egge and Swallow 2011; green anoles, *Anolis carolinensis*—Garcia et al. 2012), weaponry (e.g., Shore crabs, *Carcinus maenas*—Sneddon et al. 2000; field crickets, *Gryllus pennsylvanicus*—Judge et al. 2010), or nutritional history (e.g., burying beetles, *Nicrophorus vespilloides*—Hopwood et al. 2013). Second, RHP may be influenced by asymmetries in the motivation of opponents. Motivational asymmetries may be caused by, for example, differences between opponents’ perception of the value of the resource being fought over which may depend on factors such as ownership (e.g., jumping spiders, *Phidippus clarus*—Kasumovic et al. 2011; butterflies, *Pararge aegeria*—Bergman et al. 2010) or reproductive state (e.g., parasitoid wasps, *Goniozus nephantidis*—Stokkebo and Hardy 2000; dung roller beetles, *Canthon cyanellus cyanellus*—Chamorro-Florescano et al. 2011).

Selection on contest behavior, however, does not necessarily operate on individual fights, but rather, fighting strategies that an individual adopts over its lifetime (Kemp 2006). Like for other life-history traits, investment in contest behavior is limited by availability of resources. This can lead to trade-offs in investment, for example, between reproduction and survival, or between current and future reproduction (Stearns 1992). Life-history theory, therefore, predicts that the optimal behavioral strategy employed to mediate these trade-offs (by maximizing benefits and minimizing costs) changes over the lifetime of an individual (Stearns 1992). For example, early in life when individuals have a high chance of reproducing again, selection favors retention of resources for future reproduction and to ensure continued survival, whereas late in life, when individuals have lower residual reproductive value, selection may favor increased allocation of resources to the current reproductive attempt. Such life-history trade-offs have been suggested to underlie age-related variation sometimes seen for male aggression and contest behavior (Kemp 2006).

Although variation in motivation to win contests, which arises from differences in residual reproductive value between opponents, is likely to be important for determining age-related investment in fighting strategies, other factors may also produce age-related effects. For example, as individuals age, their fighting ability may change through changes in physical attributes such as body size or condition, as occurs in the butterfly (*Melanitis leda*) where age is negatively correlated with fighting ability, most likely due to lower energy reserves of old individuals (Kemp 2003). Conversely, in elephant seals (*Mirounga angustirostris*) fighting ability increases with age as males grow and improve condition (Haley 1994). Further, as individuals age, they may gain experience, which may be important for skill acquisition (Sharpe 2005), as well as providing information on their own fighting ability or the fighting ability of others in the population (Fawcett and Johnstone 2010). Empirical research shows that prior experience can be important in determining future contest behavior and outcomes. Most notable is research on winner and loser effects, where winners of contests are more likely to win in subsequent contests, and losers are more likely to lose in subsequent contests (reviewed in Hsu et al. 2006; Rutte et al. 2006). However, other social experiences may also influence contest behavior. For example, studies have shown that mere exposure to a potential opponent can increase aggressiveness (e.g., Argentine ants, *Linepithema humile*—Van Wilgenburg et al. 2010), and that observation of aggressive encounters between other individuals can alter future contest behavior of observers (e.g., Johnsson and Åkerman 1998; Oliveira et al. 1998). According to a recent theoretical study (Fawcett and Johnstone 2010), experience effects are expected to lead to higher levels of aggression in younger naive individuals compared with older experienced individuals. This is because younger individuals have more to gain by being more aggressive through learning about their status within the population. In addition, Fawcett and Johnstone (2010) suggest that knowledge of an individual’s status within the population influences how winner and loser effects modify future contest behavior. Younger naive individuals may be more responsive to loser effects, whereas older experienced individuals are more responsive to winner effects because of initial differences in their level of aggression (Fawcett and Johnstone 2010).

Despite the demonstrated importance of both age and social experience on contest behavior, few studies have attempted to tease apart these potentially confounding effects. Here, we manipulated both age and social experience of male burying beetles (*N. vespilloides)* to investigate their effects on contest behavior, contest outcome, and winner–loser effects. Burying beetles are ideal for studying contest behavior. Reproduction requires the use of a small vertebrate carcass that serves as a food source for both larvae and adults. Competition over these carcasses is fierce, and males (as well as females) will fight other members of the same sex to dominate access to the carcass (Eggert et al. 2008), and contest outcome is a strong determinant of reproductive strategy. Dominant males and females breed on the carcass, and subordinate individuals often adopt a satellite reproductive strategy (Bartlett 1988; Walling et al. 2008). In both males and females, the primary determinant of contest outcome appears to be body size (Otronen 1988; Eggert et al. 2008). However, experience in the form of winner and loser effects (Otronen 1990), as well as nutritional history (Hopwood et al. 2013), have also been shown to influence contest outcomes when males are matched for size.

By manipulating both male age and social experience, we aimed to investigate both the independent and interacting effects of these 2 factors on contest behavior. We predicted that if male contest behavior is governed by life-history trade-offs, old males would be more aggressive than young males. We also predicted that if males gain information about their relative competitive status through social interactions, then naive males would be more aggressive than experienced males. Additionally, we predicted that effects of male age and social experience on contest behavior would translate into differences in the likelihood of winning a contest with old and naive males more likely to win contests. Finally, according to recent theory (Fawcett and Johnstone 2010), we predicted naive males would be more responsive to loser effects, whereas experienced males would be more responsive to winner effects and that this effect would be independent of male age

## MATERIALS AND METHODS

### Stock maintenance

Experimental beetles were obtained from second-generation laboratory stock originating from 43 males and 43 females captured from Devichoys Wood, Cornwall, UK (N50°11′47″E5°7′23″) in July 2012. From this starting population, an outbred stock was maintained by mating random pairs of beetles, with each individual engaging in only 1 breeding attempt per generation. To breed, virgin pairs were placed in plastic containers (17×12×6cm) with approximately 2cm of damp soil and a mouse carcass (15–25g; sourced from Livefoods Direct, Sheffield). After dispersal, larvae were reared on a 16:8h light:dark cycle at 21°C (±1°C) in individual containers (7×7 × 4cm). Posteclosion, individuals were fed twice weekly on 2 *Tenebrio* larvae (for further details, see Head et al. 2012).

### Experimental design

We independently manipulated age and social experience of focal male beetles used in our experiment, and investigated their effects on contest behavior and outcome using a 2×2 factorial design. Thus, we set up 4 experimental treatments (old/experienced—OE, old/naive—ON, young/experienced—YE, young/naive—YN) each with 30 replicate males (*N* = 120). This allowed us to investigate the independent effects of male age and social experience, as well as their interaction on male contest behavior and contest outcome.

Males were reared over an 8 week period (September–November 2012) to give 2 age cohorts. Weekly breeding for the old-age cohort began in week 1 and weekly breeding for the young age cohort began in week 4. This meant that throughout our experiment, we were able to run contest trials for both age cohorts at the same time. Similar to previous research investigating the effects of male age on reproductive investment in burying beetles (Benowitz et al. 2013), males were allocated to 1 of 2 age treatments on eclosion. On the day of their first experimental contest, older males were 30–35 days old posteclosion, and younger males were 12–16 days old posteclosion.

Within these 2 age cohorts, we also manipulated male social experience. Naive males were maintained in isolation until their first contest. Experienced males were maintained in identical conditions to naive males until 3 days before their first contest. At this time, experienced males were placed in a clear plastic container (17×12×6cm) with 0.5cm of soil and 2 stock males (1 old and 1 young). These 2 males were randomly chosen from our stock population. This experimental setup allowed males to obtain information about their relative size and status within the population (i.e., small males would on average be smaller than the other males, large males would on average be large than the other males, and average-sized males would be on average intermediate between the other males) but did not allow males to gain direct experience of contest behavior, as social interactions between male burying beetles only escalate in the presence of a breeding resource (Pukoswki 1933). Focal males were left in these containers for approximately 24h (preliminary observations were made to ensure that focal males interacted with stock males during this period) and then kept in isolation for 48h prior to their first contest.

Prior to the establishment of our social experience treatment, we measured body size of all focal males. Pronotum width (to 0.1mm) was measured 3 times using digital calipers, and the average of these recordings was used to calculate relative pronotum width (i.e., [focal male – nonfocal male]/focal male), a measure that gives the difference in size between focal and nonfocal individuals relative to the focal individuals absolute size. Including this measure as a covariate in all analyses allowed us to control for natural variation in intrinsic fighting ability, as relative pronotum width is known to be an important predictor of contest outcome in burying beetles (Safryn and Scott 2000). Including relative size in our analyses also allowed us to test whether male age or social experience mediated the relationship between male relative size and contest outcome. Analyses conducted using the size difference between males (i.e., focal male – nonfocal male) gave similar results, and so we only present results from analyses using relative pronotum width. After size measurements were taken, focal males were given a permanent mark on the right elytron to facilitate identification during social experience and contests. Previous studies in our laboratory using marked beetles have shown that this does not affect behavior (Hopwood et al 2013), and so for logistical reasons, we only marked focal beetles and not their opponents. This method of marking is unlikely to bias our results because focal males from all treatments were marked in the same way.

### Experimental contests

Each focal male engaged in 2 experimental contests on consecutive days. This allowed us to investigate the effects of male age and social experience on contest behavior and contest outcome (analysis of the first contest), as well as how these treatments influenced winner and loser effects (analysis of second contest). For each contest, focal males were paired with opponents chosen at random from the nonfocal population. This method of pairing is preferred over size matching because it ensures randomization of variation in intrinsic fighting ability (Hsu et al. 2006), and it is more relevant to conditions beetles are likely to experience in the wild. All nonfocal males had been used in establishing our social experience treatment and so had a similar level of experience as our experienced focal males. We kept track of all individual identifications throughout the experiment to ensure that no focal male was paired with a sibling or an opponent that had been met previously, either during experimental contests or social experience treatment. Otherwise nonfocal males were allocated to contests randomly.

**Figure 1:**
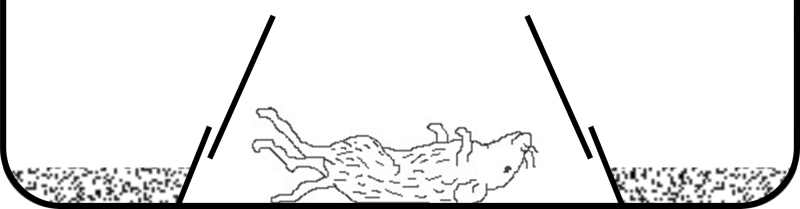
Arena for experimental contests. Escape holes remained covered until after the first interaction.

The contest arena (Figure 1) consisted of a clear perspex container (17×12×6cm) with an inner ring made from an upturned flowerpot with the base cut off (diameter: 7cm). The inner ring surrounded a small mouse carcass (19–30g, sourced from Livefoods) and was designed to promote interactions between males, but had 2 openings that allowed males to escape into the outside area if necessary. Approximately 0.5cm of soil was added to the outside area to allow individuals to burrow, and the inner ring was kept clear, in order to facilitate observation of agonistic interactions occurring on or near the carcass.

We recorded the mass of each mouse carcass (to 0.001g, Ohaus Explorer balance) to control for any effects resource value may have on contest behavior or outcome (Fawcett and Johnstone 2010). There were no effects of carcass weight on any of our response variables (all *P* > 0.473), and so it was not considered further. A fresh carcass was used for each contest.

### Behavioral observations

Both males were placed onto the carcass at the same time to avoid any effects of ownership on contest behavior or outcome as previous studies have shown that resource holders are more likely to win contests in *N. vespilloides* (Otronen 1988). Before introducing the males, the openings at the edge of the inner ring were covered to encourage pairs to interact. After initial contact, the cover was removed to allow individuals to escape into the outside area if necessary. Despite this, some pairs failed to interact throughout the initial observation period (2 pairs in the first contest, 9 pairs in the second contest).

Each pair was observed continuously for 30min after their first interaction. If no contact was made within 30min of being placed in the contest arena, the observation was terminated. The number of aggressive, submissive, and neutral interactions that the focal male engaged in was recorded (Eggert et al. 2008). All data were recorded using iObserver application version 1.1 (Skware 2011, www.skware.com) for iPad.

After the initial observation period, contest arenas (containing males) were placed into an incubator at 21°C (±1°C) and sampled every 30min to determine fight outcome. A winner was declared when one male was present on the carcass for 2 consecutive observations, and the other male was outside the inner ring. After a winner was determined, no further observations were made, and if no clear winner was seen after 3h, observations ceased.

### Statistical analysis

Only trials in which the focal male completed 2 contest trials resulting in clear outcomes were included in analyses. As a result, the original sample size of *n* = 120 per contest was reduced to *n* = 73 for each contest. Replicates were approximately evenly spread across treatments (OE = 19, ON = 19, YE = 17, YN = 18). All analyses were conducted in R version 2.13.2 (R Development Core Team, 2012).

Prior to analyzing male contest behavior, we conducted principal component analysis to obtain composite measures that best described the axes of variation in male behavior. This analysis is highly suited to the analysis of contest behavior because it accounts for the covariance structure of multiple response variables and provides composite measures of behaviors that best describe the variation in the data (Jolliffe 2002). We included data on all types of interactions (aggressive, submissive, and neutral) between focal and nonfocal males from both first and second contests. This meant that the resulting principal components were comparable across a male’s first and second contest. This analysis produced 2 vectors with eigenvalues greater than 1 (Table 1). The first principal component (PC1) described 44.0% of the variation in the data. All types of male encounters (aggressive, submissive, and neutral) loaded strongly and positively on this component, and thus, this vector represents “male encounter rate.” The second principal component (PC2) described 25.5% of the variation in the data. High values of PC2 indicate males that engage in high numbers of aggressive encounters but low numbers of submissive encounters (neutral encounters did not load strongly on this component). Thus, PC2 effectively describes variation in the ratio of aggressive to submissive encounters, and hereafter, we refer to this vector as “male aggression.”

**Table 1:** Loading of male interactions on each principal component.

	Male encounter rate (PC1)	Male aggression (PC2)
Number of fights initiated	0.543	0.632
Number of chases initiated	0.134	0.828
Number of fights received	0.877	−0.296
Number of chases received	0.834	−0.305
Number of nonescalated contacts	0.651	0.093

To investigate the effects of male age and prior social experience on these 2 behavioral vectors, as well as on contest outcome, we analyzed data from each male’s first contest using generalized linear models. Distributions of each response variable were determined from q-q plots and histograms. Male encounter rate was analyzed using a quasi-Poisson error distribution, and male aggression was analyzed using a Gaussian distribution. Contest outcome (i.e., winner or loser) was analyzed using a binomial distribution. Each model included male age and social experience treatment as fixed effects, relative male size (i.e., [focal male pronotum width – nonfocal male pronotum width]/focal male pronotum width) as a covariate, and all 2-way interactions (Briffa et al. 2013). Minimal adequate models were obtained by stepwise elimination of nonsignificant terms (Crawley 2007). We also analyzed our data including only contests where males differed in size by less than 0.5mm because motivational effects are likely to play a greater role in individuals that are closely matched in size. These analyses gave qualitatively similar results to the full dataset, and so we present the results from our full dataset only here. The similarity of these results attests to the robustness of our conclusions. Furthermore, we investigated whether there were quadratic effects of relative size on contest behavior or outcome. As there were none, the results are not presented here.

To investigate the effects of male age and prior social experience on winner and loser effects, we analyzed data from each male’s second contest using generalized linear models. We used a similar approach as that outlined above for analyzing a male’s first contest except that we also included the outcome of a male’s first contest as a fixed factor. This allowed us to determine if there were any winner or loser effects (i.e., an effect of first contest outcome on any of our response variables), as well as whether male age or social experience mediated these winner–loser effects (i.e., any significant effects of the interactions between these treatments and outcome of a male;s first contest on any of our response variables).

## RESULTS

### Effects of age, experience, and relative male size on contest behavior

Contrary to expectation, male age did not influence contest behavior. There were no main effects of age on either of our behavioral variables (male encounter rate: *F*_(1,70)_ = 2.529, *P* = 0.1163; male aggression: *F*_(1,69)_ = 0.039, *P* = 0.844). Male age did not influence contest behavior through any interactions with other terms included in the models (all interaction terms removed from the models at *P* > 0.219).

**Figure 2:**
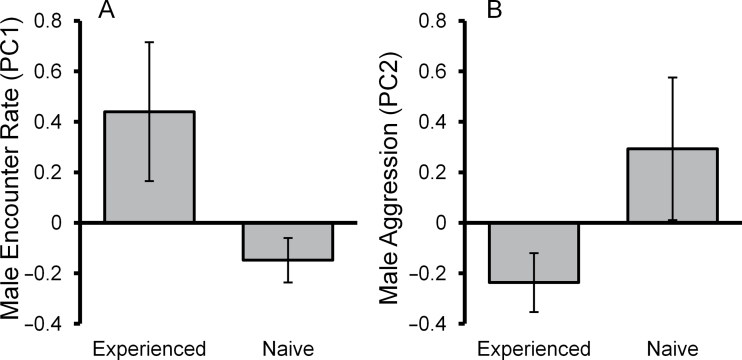
Effect of social experience on male contest behavior during a male’s first contest. (A) Male encounter rate and (B) male aggressive behavior. Mean ± standard error.

Social experience on the other hand did influence contest behavior. However, this was not via a significant effect on male aggression (*F*_(1,71)_ = 3.010, *P* = 0.087) as was predicted, but rather via a significant effect on male encounter rate (*F*_(1,71)_ = 4.942, *P* = 0.029). Males with prior social experience had higher encounter rates than naive males during their first contest (Figure 2). All interaction terms involving social experience were removed from the models (*P* > 0.077).

Neither male aggression nor male encounter rate were related to male relative size (aggression: *F*_(1,70)_ = 0.924, *P* = 0.339; encounter rate: *F*_(1,69)_ = 1.285, *P* = 0.261).

### Effects of age, experience, and relative male size on contest outcome

**Figure 3:**
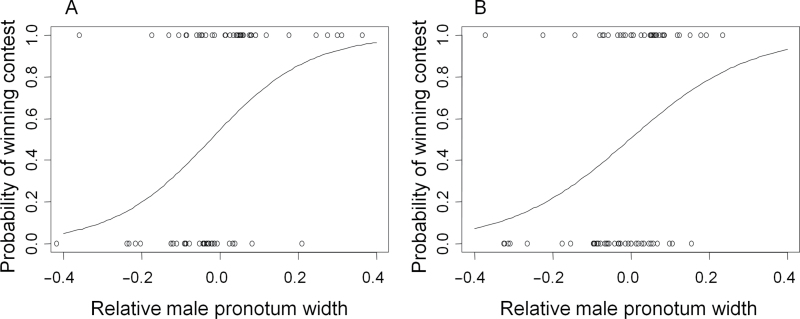
Logistic relationships showing the effect of relative pronotum width ([focal male – opponent]/focal male) on contest outcome. (A) A male’s first contest and (B) a male’s second contest. Mean ± standard error.

Male relative size was the only term in our model that had a significant effect on the outcome of a male’s first contest (χ^2^_(1,72)_ = 13.178, *P* < 0.001 (Figure 3a). Neither male age nor social experience had significant main effects on the outcome of a male’s first contest (male age: χ^2^_(1,70)_ < 0.006, *P* = 0.939; social experience: χ^2^_(1,71)_ = 1.533, *P* = 0.216), nor did they influence contest outcome through any interaction effects (all interactions removed from the model at *P* > 0.163).

### Effects of age, experience, and relative size on winner–loser effects

We found very little evidence for either winner or loser effects in this study. The outcome of a male’s first contest did not influence the outcome of a males second contest (χ^2^_(1,71)_ = 0.936, *P* = 0.333). We also found no evidence that winner or loser effects mediated the relationship between male relative size and contest outcome (i.e., the interaction between relative pronotum width and outcome of first contest was not significant: χ^2^_(1,65)_ = 0.129 *P* = 0.7187). Further, we found no evidence that male age or experience influenced the outcome of a males second contest (male age: χ^2^_(1,70)_ = 0.118, *P* = 0.739; social experience: χ^2^_(1,69)_ = 0.025, *P* = 0.875) or that these factors mediated winner or loser effects (i.e., interactions involving these terms were dropped from the model at *P* > 0.212). The only term included in our model that predicted the outcome of a male’s second contest was relative size (χ^2^_(1,72)_ = 8.801, *P* = 0.003).

**Figure 4:**
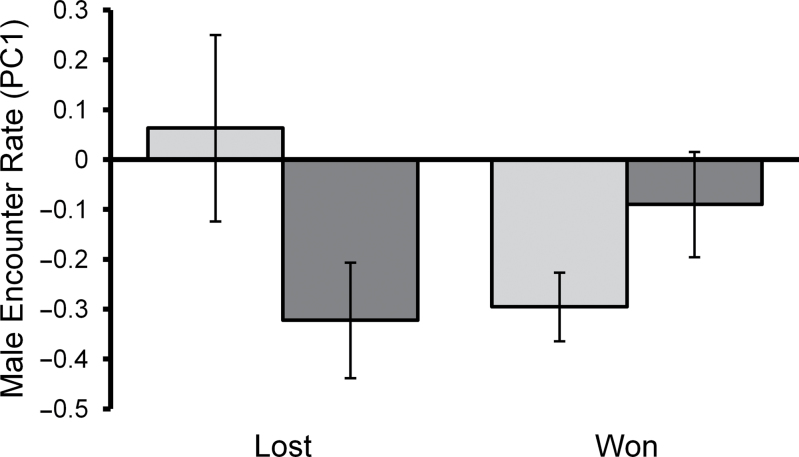
The effects of first contest outcome and social experience treatment on male encounter rate during a male’s second contest. Light bars represent males with prior social experience and dark bars represent naive males. Mean ± S.E.

Male contest behavior in the second contest was, however, influenced by the outcome of a male’s first contest and by prior social experience. These factors interact to influence male encounter rate (interaction: *F*_(1,66)_ = 5.522, *P* = 0.022; fight 1 outcome: *F*_(1,66)_ = 3.986, *P* = 0.050; social experience: *F*_(1,66)_ = 4.366, *P* = 0.041) and similar to results from a males first contest, do not influence male aggression. Experienced males that won their first contest had lower encounter rates than experienced males that had lost their first contest, whereas for naive males, there was no difference in behavior whether they won or lost their first contest (Figure 4). For male encounter rate, all other terms were dropped from the model (all *P* > 0.176). For male aggression, all terms were dropped from our final model (all *P* > 0.154) leaving only the intercept.

## DISCUSSION

Life-history theory predicts that individuals should increase investment in reproduction as their residual reproductive value decreases. In animals that compete for breeding resources, this could lead to increased aggression as individuals age (Kemp 2006). However, as individuals age, they also gain information on their relative status in the population, which may influence whether they are likely to engage in costly contest behavior and thus whether they are likely to dominate breeding resources. Recent theory regarding the effects of experience on contest behavior suggests that as an animal ages and gains experience, it should become less aggressive (Fawcett and Johnstone 2010). This is because older individuals have nothing to gain from escalating a contest they are unlikely to win, whereas younger individuals gain by learning about their relative status within the population. Here, we tease apart the often confounded effects of male age and social experience on male fighting behavior to test how these factors influence male contest behavior and outcomes. Contrary to our predictions, we found that male age did not influence contest behavior or contest outcome. In contrast, social experience did influence contest behavior; however, this was via effects on male encounter rates rather than predicted effects on male aggression, and these differences did not affect the outcome of contests. By far the strongest predictor of whether a male won or lost a contest was his size relative to the size of his opponent.

### Effects of age on contests

In accordance with life-history theory, we predicted that older males would be more aggressive and more likely to win contests over breeding resources. Contrary to these predictions, we found no evidence that age influenced contest behavior or outcome. Our age treatment was specifically designed to investigate the influence of residual reproductive value on contest behavior while controlling for other potential age-related factors. Residual reproductive value is a measure of potential future reproduction, which may depend on both the resources an individual has left for reproduction, as well as the potential opportunities an individual has left for reproduction (Roff 1992). It is possible that because our beetles were kept isolated in controlled environments for much of their lives, their energy expenditure was low, possibly reducing differences in residual reproductive value between old and young males and thus the likelihood of detecting age effects. However, previous studies using similar age treatments have shown that male burying beetles do show terminal investment in both mating and parental care behavior (Benowitz et al. 2013). Thus, we believe that if there was a benefit to males investing terminally in contest behavior, we would have detected it here. So the question that needs to be answered is why do male burying beetles terminally invest in mating and parental care but not in contests?

Our results show that not only was there no difference between young and old males in their ability to win a contest (i.e. no effect of age on contest outcome), but there was also no effect of male age on male motivation to win a contest (i.e. no effect of age on contest behavior). As for many species (reviewed in Arnott and Elwood (2009)) we show that size is a strong predictor of the likelihood of winning a contest in *N. vespilloides*. Only in experiments where investigators have size matched opponents have effects of other factors, such as nutritional state (Hopwood et al. 2013) and fighting experience (Otronen 1990), been found. Also, body size in wild burying beetles is highly variable (Otronen 1988), and as such it is unlikely that burying beetles would ever encounter situations where modifying their contest behavior is likely to change the outcome of a contest. Furthermore, burying beetles have alternative reproductive tactics and males who are unable to dominate a breeding resource may still obtain reproductive success by becoming satellites. For these reasons, selection on plasticity in contest behavior is may be weak and older males that are smaller than their opponents may be better off increasing investment in satellite behavior rather than increasing investment in contest behavior, even if they are unlikely to have further opportunities for reproduction.

Previous studies that have shown age-related variation in contest behavior and likelihood of winning contests have done so in species where age is related to fighting ability (Jennings et al. 2010) or where motivation has been shown to be an important contributor to RHP (Kemp et al. 2006). As residual reproductive value is likely to influence contest behavior via effects on motivation rather than fighting ability, these studies provide further, albeit indirect, evidence that age effects on contest behavior that result from variation in residual reproductive value may be weak when fighting ability plays a prominent role in determining contest outcome.

### Effects of experience on contests

We examined how 2 types of prior experience influence contest behavior and outcome. First, we manipulated male social experience prior to any contests to investigate whether males gain information about their relative status in the population through interactions with other beetles. Second, we investigated whether fighting experience led to winner or loser effects. We found that neither social experience nor fighting experience influenced the likelihood that a male would win a contest, but both types of experience did influence contest behavior via effects on male encounter rates.

Winner and loser effects have been reported in many taxa (including the burying beetle, *Nicrophorus humator*—Otronen 1990) and are primarily thought to arise due to changes in an individual’s perception of their relative competitive status within a population (reviewed in Hsu et al. 2006; Rutte et al. 2006). However, winner and loser effects could also be produced by other mechanisms. For instance, winners may gain access to resources that allow for increased fighting ability (Kasumovic et al. 2010). Likewise, the act of obtaining a breeding resource may set in motion physiological changes in reproductive state that increase the perceived value of a resource in subsequent contests (Goubault and Decuignière 2012). Similarly, loser effects may be caused by a reduction in an individual’s fighting ability through injury or reduced energy reserves (Rutte et al. 2006).

Few studies have investigated the role that social experiences other than fighting have on contest behavior (Van Wilgenburg et al. 2010). Social experience has previously been shown to be important in developing the perception of relative status of individuals in the population. For example, in the context of mating, individuals may gain information on their own relative quality (Holveck and Riebel 2010) or learn about the relative quality of potential mates (Kozak and Boughman 2009) from prior (nonsexual) social experiences. Also, individuals that have been kept in isolation prior to contests are often more aggressive than those that have been allowed social interactions (reviewed in Hsu et al. 2006). Thus, if winner and loser effects are produced by changes in perceived relative competitive status within the population, other social experiences should also play a role in determining contest behavior, particularly in species where contest behavior is costly and where other cues to assess fighting ability exist, as may be the case when morphology correlates strongly with fighting ability.

The effects of experience on contest behavior that we see here do not support the idea that male burying beetles gain information on their relative competitive status through either social experience per se or through fighting experience more specifically. According to theory (Fawcett and Johnstone 2010), experienced individuals with information about their relative status in the population should be less aggressive than naive individuals and should be less responsive to winner effects than naive individuals. We found neither of these effects. This suggests that previous winner–loser effects found in burying beetles (Otronen 1990) may be due to changes in actual fighting ability, resulting perhaps from injury, rather than changes in perceived fighting ability. Or that the assessment strategy employed by males does not allow them to assess their own absolute RHP during interactions and then arrive at an estimate of their relative RHP in subsequent contests. Further studies investigating how experience alters subsequent contest behavior in the absence of potentially confounding physiological effects of winning and losing are needed to test whether self-perception of fighting ability has a general role to play in determining contest behavior.

If the effects of experience on behavior that we see here are not due to changes in perceived likelihood of winning contests, what could be their cause? The fact that both fighting experience and social experience per se influence the same behavior suggests it is not fighting itself that individuals are responding to but rather the fact that they have interacted with other individuals. Social experience is known to mediate many behavioral and physiological processes in animals. For instance, studies have shown that prior social experience can influence boldness (Frost et al. 2007; Edenbrow and Croft 2013) or stress hormones (Sachser et al. 2013). In addition, prior social experience may influence how individuals are perceived by others (Ruploh et al. 2013). Given the complexity of the possible effects of prior social experience on behavior, further investigation and manipulative experiments are required before we can interpret the specific effects that we see here.

An alternative explanation for our results is that rather than manipulating male self-perception of fighting ability, our social experience treatment may be manipulating male perception of population density. Previous work suggests that aggression is likely to be greatest at intermediate population densities (reviewed in Knell 2009). This is because under low population densities, males may redirect investment away from aggression toward mate searching, and under high population densities, males may redirect investment from aggression toward alternative mating strategies (Knell 2009). Our results show that experienced males had higher encounter rates during their first contest than naive males. This suggests that high encounter rates are not due to a switch toward mate searching resulting from male perception of low population density. To determine whether perception of population density influences contest behavior in this system would require further studies manipulating the number or frequency of individuals that a male encounters prior to contests.

## CONCLUSIONS

Our male age and social experience treatments were designed to manipulate male residual reproductive value and perception of relative fighting ability within the population (respectively). Both of these factors are expected to influence male motivation to escalate contests rather than their ability to win contests. Motivation has been suggested to influence contest behavior and outcome in many species (Bergman et al. 2010). However, there are few cases where effects on fighting ability can be ruled out altogether. Our results suggest that male motivation has little (if any) affect on contests in male *N. vespilloides*. We suggest that this is likely because body size is a strong predictor of success in competitive situations and high natural variation in this trait means that selection on factors that influence contest outcomes via changes in motivation is likely to be weak. Furthermore, in this species males may adopt alternative reproductive tactics that allow them to gain reproductive success even when they do not dominate a carcass. Our study highlights the need to place contest studies in an ecological context if we are to understand how variation in contest behavior evolves. In species where morphological traits (e.g., body size or weapon size) are strong predictors of fighting ability and where there is high variation in the size of such morphological traits in natural populations, then selection on factors that influence the motivation of individuals to engage in contests is likely to be weak. Thus, the only factors that are likely to influence contest outcomes are those that influence fighting ability itself.

## FUNDING

Natural Environment Research Council (UK) grant (NG/H022805/1) to N.J.R.
